# A Rare Cause of Acquired Non-Wilsonian Hepato-Cerebral Degeneration in a Middle-Aged Woman

**DOI:** 10.7759/cureus.71361

**Published:** 2024-10-13

**Authors:** Mahika Kamat, Karan Muzumdar, Muhammedameen Noushad, Shakya Bhattacharjee, Jigar Vora

**Affiliations:** 1 Department of Acute Medicine, University Hospitals Plymouth NHS Trust, Plymouth, GBR; 2 Department of Neurology, University Hospitals Plymouth NHS Trust, Plymouth, GBR

**Keywords:** basal ganglia, chronic liver disease, dystonia, manganese, parkinsonism

## Abstract

Manganese is an essential trace mineral that has a vital role in maintaining various body functions. Manganese toxicity, referred to as Manganism, causing parkinsonism is a well-known phenomenon that occurs secondary to chronic environmental and occupational exposure to manganese. Patients with underlying chronic liver disease are also susceptible to the toxic accumulation of manganese since it essentially undergoes biliary excretion. At high serum concentrations, manganese can cross the blood-brain barrier and cause neuronal damage. We report an interesting case of a middle-aged woman with underlying chronic liver disease presenting to the medical admissions unit with features suggestive of parkinsonism, dystonia, and cerebellar dysfunction that developed secondary to manganese deposition in the basal ganglia of the brain. The report discusses important radiological features and also lays special emphasis on early recognition, clinical, radiological, and biochemical correlation, and early intervention.

## Introduction

Parkinsonism is the clinical term for a disorder with prominent bradykinesia and the presence of extrapyramidal signs and symptoms to a variable degree. The pathophysiology involves the degeneration of nigro-striatal dopaminergic pathways with neuronal loss and reactive gliosis in the substantia nigra commonly found at autopsy [1]. Dystonia is a movement disorder characterized by abnormal repetitive movements or postures owing to sustained or intermittent muscle contractions [2]. Manganese is an essential trace element that can cross the blood-brain barrier and act as a neurotoxin at higher serum levels [3]. We present an interesting case of a middle-aged female with features of mild parkinsonism, dystonia, and cerebellar dysfunction that were attributed to the toxic accumulation of manganese in the basal ganglia of the brain.

This article was previously presented as a virtual poster at the XXVI World Congress of Neurology (WCN 2023) held from October 15-19, 2023.

## Case presentation

**Figure 1:**
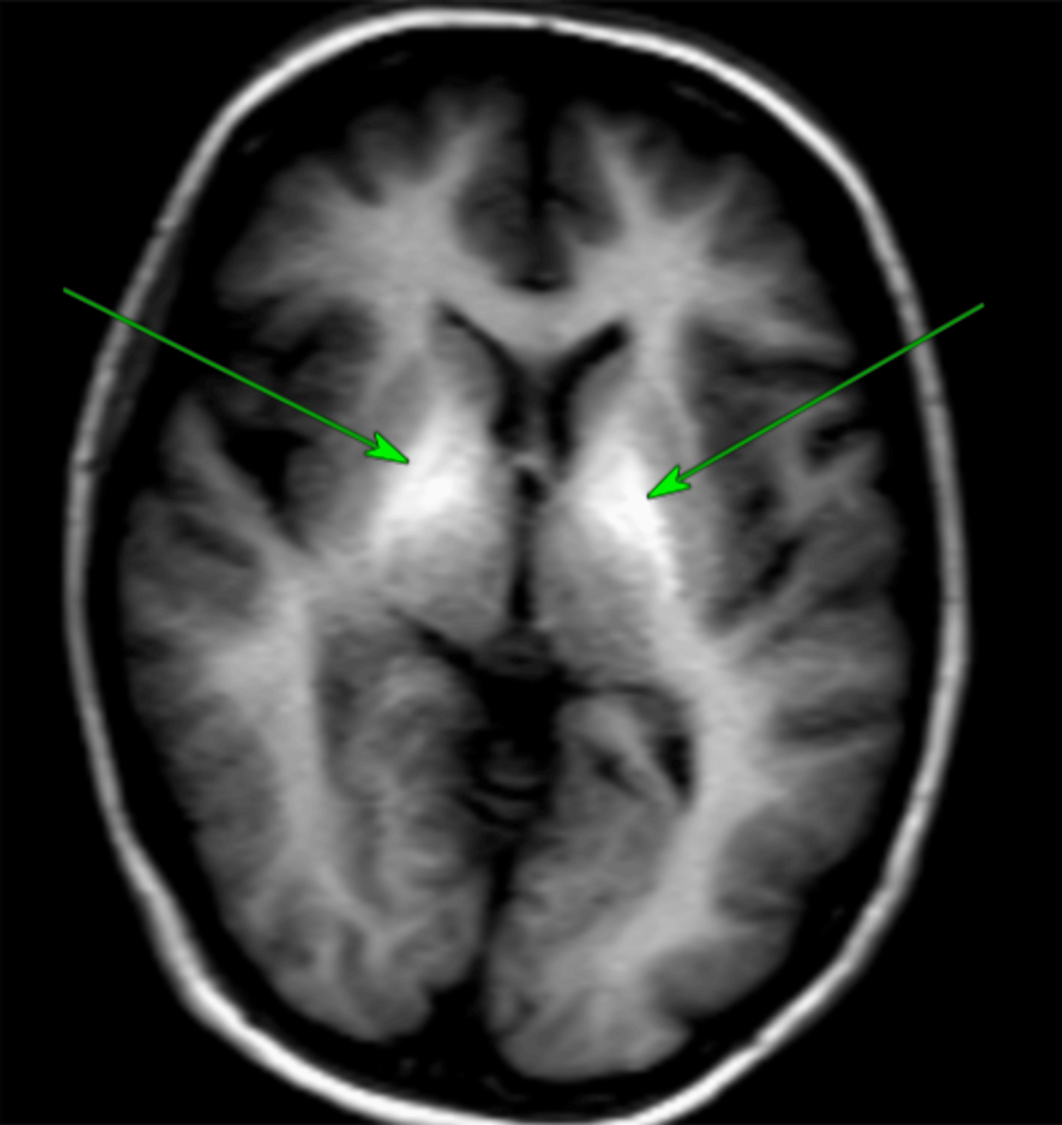
T1-weighted axial MRI of the brain The two arrows indicate the areas showing bilateral symmetric T1 hyperintensity in the globus pallidus

**Figure 2:**
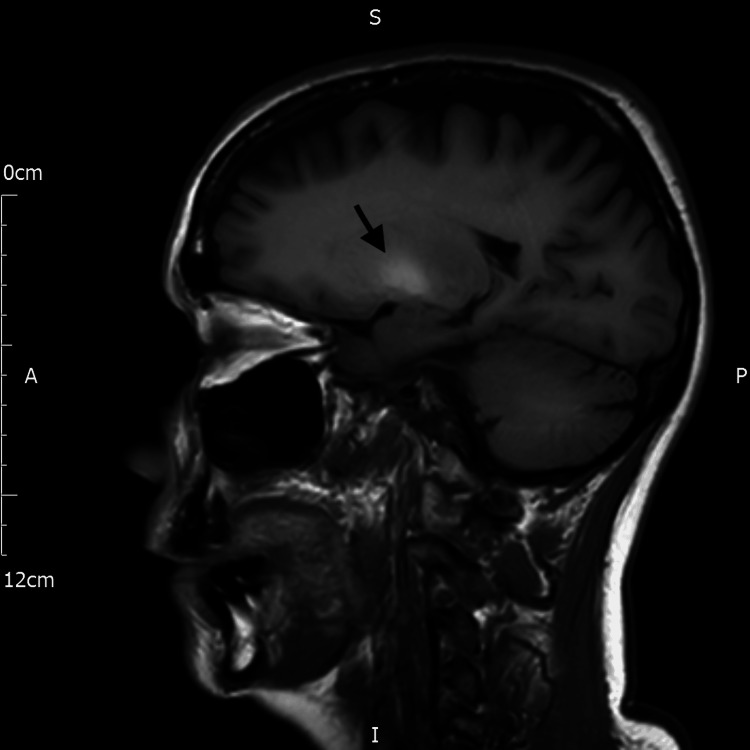
T1-weighted sagittal MRI of the brain The arrow indicates high signal intensity in the region of the basal ganglia

**Figure 3:**
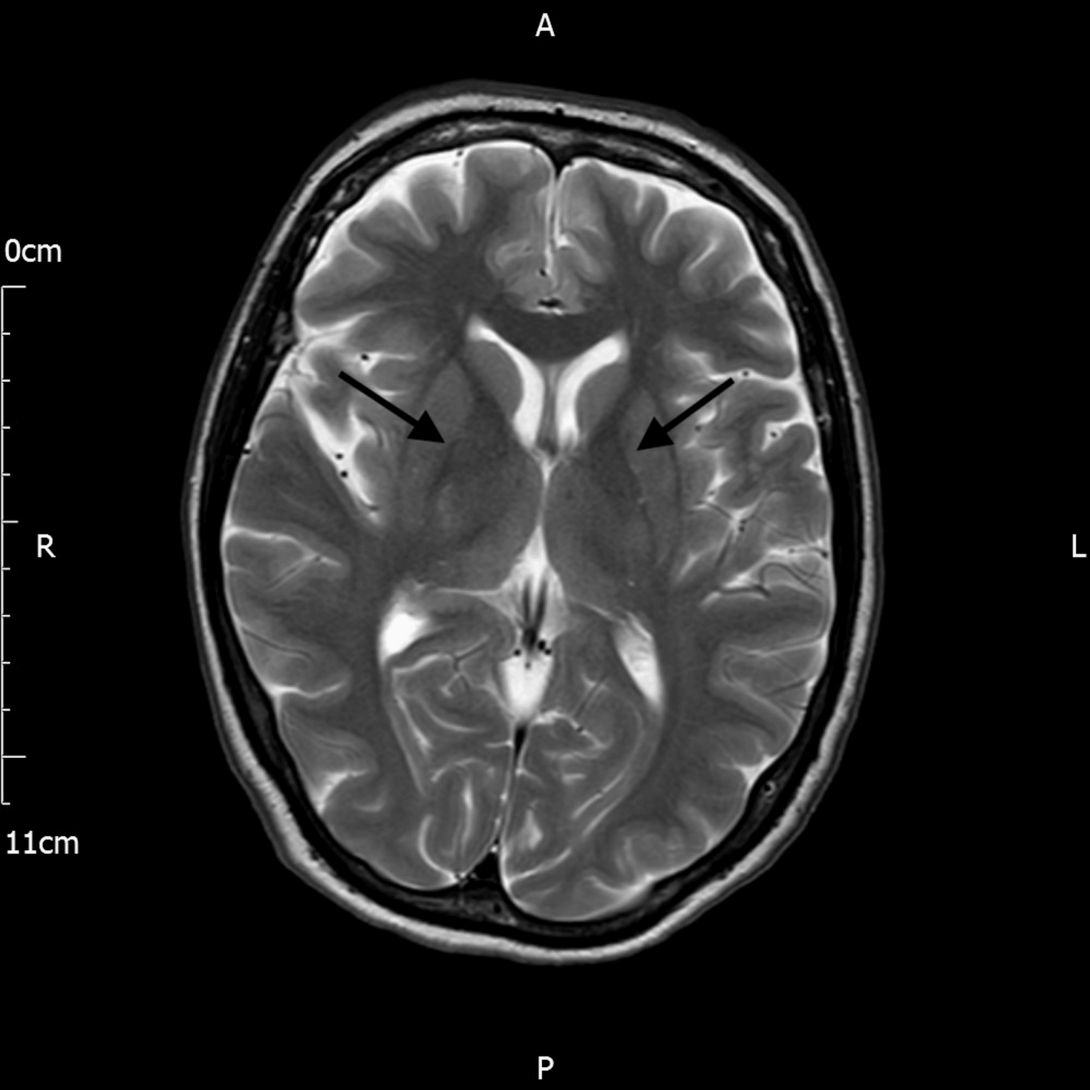
T2-weighted axial MRI of the brain The arrows indicate the absence of hyperintensity in the region of the basal ganglia

**Figure 4:**
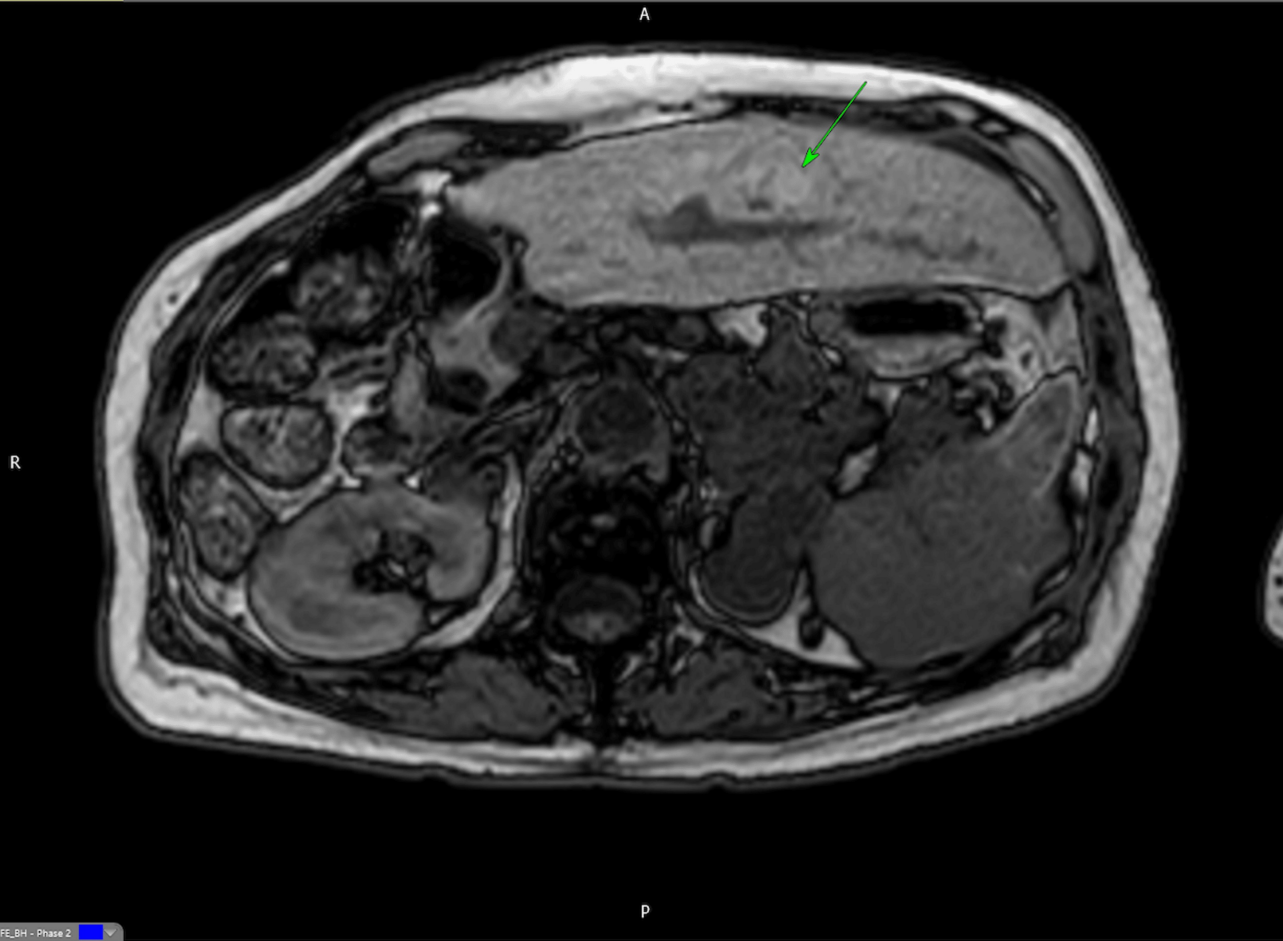
T1-weighted MRI of the liver The arrow indicates the area with the intrinsic high T1 signal

A 58-year-old female presented to the medical admissions unit with a seven-day history of loss of balance, lethargy, reduced oral intake, and difficulty with activities of daily living. There was no history of fever, cough, coryza, dizziness, acute shortness of breath, abdominal pain, vomiting, diarrhea, urinary symptoms, or recent trauma. She had a background of congenital hepatic fibrosis, portal hypertension, bowel cancer, essential hypertension, dilated cardiomyopathy, progressive heart failure, alcohol use disorder, and depression. She was previously known to the hepatology team and was undergoing evaluation for hepatic transplantation. She lived with her partner and was previously independent with her daily activities. However, she now reported poor exercise tolerance. Her regular medications included rifaximin and lactulose which were commenced during a previous hospital admission for hepatic encephalopathy. On examination, her vital signs including blood pressure, heart rate, body temperature, oxygen saturation, and respiratory rate were within normal limits. She was alert and orientated to time, place, and person. She appeared frail and had dry mucus membranes, and there was evidence of scleral icterus but no pallor, cyanosis, finger clubbing, or lymphadenopathy was noted. Cardiovascular, respiratory, and abdominal examinations were unremarkable. On neurological examination, she had bilateral postural and action tremors, upper limb rigidity with dystonic posturing, and bradykinesia. However, no resting tremors were noted. She was mildly dysarthric and had a broad-based ataxic gait and evoked nystagmus on the left and right lateral gaze. Her muscle bulk and reflexes were globally preserved and she had bilaterally down-going plantar reflexes. Kayser-Fleischer rings were not seen on external ocular examination. Given the significant findings on neurological examination, magnetic resonance imaging (MRI) of the brain was carried out and this demonstrated T1 hyperintensity in the region of the basal ganglia (Figures 1, 2) without corresponding T2 hyperintensity (Figure 3). There was no evidence of cerebellar atrophy. MRI of the liver that was previously carried out as part of her evaluation for hepatic transplantation also demonstrated an area with an intrinsic high T1 signal (Figure 4). Initial blood tests revealed a normal full blood count and C-reactive protein. Urea was mildly elevated at 10 mmol/L (2.5-7.8 mmol/L) but creatinine, eGFR, and electrolytes were within normal limits. Liver function tests were abnormal with a total bilirubin of 83 umol/L (1-20 umol/l) of which conjugated bilirubin was 16 umol/L (0-8.6 umol/L). Aspartate transaminase (AST) and alkaline phosphatase (ALP) were both elevated at 65 IU/L (5-34 IU/L) and 207 IU/L (30-130 IU/L) respectively. She was also noted to have a deranged clotting profile with an elevated prothrombin time (PT) of 23.7 seconds (12.5-15.5 seconds) and an activated partial thromboplastin time (APTT) of 44.1 seconds (25.4-34 seconds). Subsequently, a decision was made to conduct a thorough biochemical evaluation to identify a cause for her movement disorder. She was noted to have a slightly elevated ammonia level of 80 umol/L (18-72 umol/L). Tumor markers including AFP, CEA, and CA 19-9 were within normal limits and a viral screen for hepatitis, CMV, HIV, EBV, and toxoplasma was negative. A comprehensive autoimmune panel consisting of anti-nuclear, anti-smooth muscle, anti-mitochondrial, anti-Ri, anti-Hu, anti-purkinje cell, anti-gastro-parietal and anti-liver-kidney microsomal antibodies was also negative. While serum alpha-1 antitrypsin, zinc, copper, and ceruloplasmin levels were within normal limits, a screen for other trace elements revealed elevated serum manganese levels of 654 nmol/L (70-120 nmol/L). Given her background of chronic liver disease, she was suspected to have developed features of parkinsonism, dystonia, and cerebellar syndrome secondary to manganese toxicity. The findings noted in MRI scans of the brain and liver were initially attributed to the toxic accumulation of manganese within these organs. However, the areas with a high T1 signal on MRI liver could also represent regenerative nodules, given the background of congenital hepatic fibrosis and the absence of signal loss in the chemical shift sequence. She was commenced on a trial of co-careldopa to help with her symptoms of parkinsonism and was discussed at the multi-disciplinary meeting for a liver transplant. She was discharged from the hospital and referred to the outpatient movement disorder clinic in four weeks. Unfortunately, given her poor physiological reserve and multiple medical co-morbidities, she was eventually deemed unfit for transplant surgery and died as a result of decompensated liver disease three months later.

## Discussion

Patients with chronic liver disease manifest a high incidence of pallidal signal hyperintensity on T1-weighted MRI as a result of manganese deposition in the brain. This occurs mainly in the putamen, globus pallidus and occasionally in the dentate nucleus, frontal and occipital cortex [4-6]. The deposition of manganese in the basal ganglia and midbrain noticed in our patient was rarely reported. Generally, the pars reticulata of the substantia nigra of the midbrain is affected and the pars compacta of the midbrain is spared [7]. This relative sparing of substantia nigra can explain the presence of mild parkinsonism contrary to idiopathic Parkinson’s disease. Elevated levels of manganese were also found in cerebrospinal fluids of these patients. This was suggestive of widespread manganese accumulation. The autopsy findings of patients who died as a result of chronic liver disease-induced hepatic coma reveal a 2 to 7-fold increase of pallidal manganese and a concomitant loss of dopamine D2-receptor binding sites [5]. Though manganese deposition is a major cause of pallidal hyperintensity on T1-weighted MRI, the other known causes are the deposition of copper, iron, methemoglobin, melanin, proteinaceous materials, and fat products. The degree of MRI change often correlates with blood manganese levels and the presence of clinical symptoms. The important differentials that could explain the high intrinsic signals on T1 weighted MRI of the liver in this case would be mineral deposition, regenerative nodules, dysplastic nodules, hepatocellular carcinoma (HCC), and focal nodular hyperplasia. Subtracted images (without the intrinsic high T1 signal) did not show any enhancement, making typical HCC less likely. Furthermore, there was no evidence of restricted diffusion, which meant that the cells being examined did not have radiological evidence of high mitotic index. Chronic manganese intoxication in the absence of liver disease was also found in cases of patients receiving total parenteral nutrition and in those employed as welders and alloy industry workers. The common neurological signs and symptoms reported with manganese toxicity are those of parkinsonism, dystonia, myoclonus, and cognitive decline. Asymmetric resting tremors are less common in manganese-induced parkinsonism and cognitive involvement may occur early. Features of parkinsonism secondary to manganese deposition noted in welders and drug addicts would be less responsive to levodopa as the underlying mechanism is pallidal injury with relative sparing of the nigro-striatal pathway. Liver transplantation may normalize the pallidal MR signals and serum manganese level. However, the T1 hyperintensity may take up to one year to resolve after successful liver transplantation.

## Conclusions

Manganese toxicity may often be overlooked as a cause of early-onset parkinsonism and hepato-cerebral degeneration. In patients with chronic liver disease presenting with features of hepato-cerebral degeneration, it is imperative to consider metabolic causes early as these patients can be potential candidates for life-saving interventions. Robust studies involving randomized controlled trials need to be carried out to determine the efficacy of medical treatments such as levodopa and EDTA chelation. At present, liver transplantation is the definitive treatment in these patients.
